# Editorial: Mechanisms of neuroinflammation and inflammatory neurodegeneration in acute brain injury

**DOI:** 10.3389/fncel.2015.00300

**Published:** 2015-08-05

**Authors:** Arthur Liesz, Christoph Kleinschnitz

**Affiliations:** ^1^Institute for Stroke and Dementia Research, Klinikum der Universität MünchenMunich, Germany; ^2^Munich Cluster for Systems Neurology (SyNergy)Munich, Germany; ^3^Department of Neurology, University Hospital WürzburgWürzburg, Germany

**Keywords:** stroke, intracerebral hemorrhage, traumatic brain injury, neuroinflammation, leukocytes

The current research topic and eBook “Mechanisms of neuroinflammation and inflammatory neurodegeneration in acute brain injury” was initiated as a reaction to the rapidly expanding literature on inflammatory mechanisms in the pathophysiology of acute brain injuries. The scope of this compilation of reviews, opinion, and original research articles was to give a broad overview of the diverse cellular compartments and mechanisms involved in the inflammatory response to brain tissue injury.

Although a specific aspect of the pathophysiology of acute brain injuries, the immune system interacts in highly complex as well as diverse mechanisms with the damaged brain.

On one side acute brain lesions, such as brain ischemia, hemorrhage or traumatic injury, induce a local neuroinflammatory reaction, wherein microglial cells represent the local immune cells (Benakis et al., 2015; Lourbopoulos et al., 2015). This local inflammatory response has a major impact on outcome with differential effects during the phases of post-stroke lesion evolution and recovery (Shichita et al., 2014). Intriguingly, besides abundant evidence on post-stroke neuroinflammation immunological mechanisms similar mechanisms are also observed in traumatic brain injuries (Schwarzmaier and Plesnila, 2014), intracerebral hemorrhage (Mracsko and Veltkamp, 2014) and even ethanol-induced neurotoxicity (Alfonso-Loeches et al., 2014; Sokolowski et al., 2014) or direct application of exogenous pathogens (Gullo et al., 2014) with functional consequences for neuronal outcome. Moreover, Gauberti et al. (2014) present an overview on state-of-the-art molecular magnetic resonance imaging of neuroinflammatory markers. In recent years also the molecular pathways and effector molecules of inflammation-induced neurotoxicity after acute injuries have been investigated in great detail: Murray et al. (2015) describe in their review the prominent role of the pro-inflammatory cytokine IL-1, Orsini et al. (2014) give an overview on the complement system in neuroinflammation, while Albert-Weissenberger et al. (2014a) focus on the contribution of the kallikrein-kinin system in traumatic brain injury and Zhao et al. (2014) review the current knowledge on programed death-1/programed death ligand signaling. In addition to the activation of local inflammatory pathways in the injured brain, invasion of peripheral immune cells to the brain is a critical step in secondary neuroinflammation. Gelderblom et al. (2014) review the role of gdT cells as a pro-inflammatory invariant T cell subpopulation recruited to the injured brain. In contrast, Urra et al. (2014) discuss potential mechanisms of antigen-specific autoimmunity after acute brain injury. In addition, the original research article by Kim et al. (2014) underlines that the cellular immune response to ischemic brain injury might differ substantially between commonly used mouse strains.

In addition to an overview and discussion of basic mechanisms and involved pathways in secondary neuroinflammation after acute brain injury, our research topic also contains several reviews and original articles on novel therapeutic approaches to modulate the immune response. Rissiek et al. (2014) introduce nanobodies as a novel tool for targeting neuroinflammation. Brunkhorst et al. (2014) provide an overview on the promising approach of blocking cellular neuroinflammation with Fingolimod. Bodhankar et al. (2014) review the current literature on targeting the PD-L1 and PD-L2 pathways. The original article by Mouihate (2014) reports a novel role for hormonal replacement therapy in neuroinflammation and the original article by Albert-Weissenberger et al. (2014b) the use of C1-inhibitors in a cortical cryolesion model. Dotson et al. (2014) have tested the use of recombinant TCR ligand with differential effects in young and old mice (see also commentary by Pennypacker, 2014).

In summary this research topic gathered contributions from the leading laboratories working in the field of secondary neuroinflammation after brain injury with nearly 100 authors from 4 continents. We are confident that this compilation covers most established and emerging research questions in this specific research field and presents an up-to-date overview on inflammatory mechanisms and drug targets in acute brain injuries.

## Conflict of interest statement

The authors declare that the research was conducted in the absence of any commercial or financial relationships that could be construed as a potential conflict of interest.
